# SPARC: a potential target for functional nanomaterials and drugs

**DOI:** 10.3389/fmolb.2023.1235428

**Published:** 2023-07-28

**Authors:** Shan Jiang, Hui-Feng Sun, Shuang Li, Ning Zhang, Ji-Song Chen, Jian-Xin Liu

**Affiliations:** ^1^ School of Pharmacy, Heilongjiang University of Traditional Chinese Medicine, Harbin, China; ^2^ School of Pharmaceutical Sciences, Department of Rehabilitation and Healthcare, Hunan University of Medicine, Huaihua, China; ^3^ College Pharmacy, Jiamusi University, Jiamusi, China; ^4^ School of Pharmaceutical Sciences, University of South China, Hengyang, China; ^5^ Institute of Innovation and Applied Research in Chinese Medicine, Hunan University of Chinese Medicine, Changsha, China

**Keywords:** SPARC, rheumatoid arthritis, target, tumors, functional nanomaterials

## Abstract

Secreted protein acidic and rich in cysteine (SPARC), also termed osteonectin or BM-40, is a matricellular protein which regulates cell adhesion, extracellular matrix production, growth factor activity, and cell cycle. Although SPARC does not perform a structural function, it, however, modulates interactions between cells and the surrounding extracellular matrix due to its anti-proliferative and anti-adhesion properties. The overexpression of SPARC at sites, including injury, regeneration, obesity, cancer, and inflammation, reveals its application as a prospective target and therapeutic indicator in the treatment and assessment of disease. This article comprehensively summarizes the mechanism of SPARC overexpression in inflammation and tumors as well as the latest research progress of functional nanomaterials in the therapy of rheumatoid arthritis and tumors by manipulating SPARC as a new target. This article provides ideas for using functional nanomaterials to treat inflammatory diseases through the SPARC target. The purpose of this article is to provide a reference for ongoing disease research based on SPARC-targeted therapy.

## Introduction

Targeted therapy of inflammation and tumor is the main focus of the current research. To date, there is no perfect treatment plan for rheumatoid arthritis, which is a well-known immunodeficiency inflammatory disease (Watanabe et al., 2022). With the development and synthesis of functional nanomaterials, serum albumin, such as human serum albumin (HSA), has become a popular material used in the cure of rheumatoid arthritis. The powerful affinity between serum albumin and secreted protein acidic and rich in cysteine (SPARC) has become a huge driving force for drug-targeted therapy (Liu et al., 2019). In tumor treatment, functional nanomaterials have achieved certain results by manipulating SPARC-targeted therapy. The specific binding between HSA and nab-paclitaxel realizes the purpose of drug-dependent release and precise targeted therapy of cancer (Yardley, 2013). The high binding of HSA to SPARC realizes the targeted aggregation of paclitaxel in tumor lesions. The discovery of biomimetic drug delivery of functional nanomaterials *in vivo* by manipulating SPARC significantly alleviates rejection (Lin et al., 2016). This article will introduce the regulatory mechanism and research progress of SPARC in rheumatoid arthritis and tumors in detail. Based on the pathological phenomenon of high-level expression of SPARC in rheumatoid arthritis and tumors, it is more feasible for functional nanomaterials to deliver drugs directly to the lesion.

This article reviews the regulatory mechanism and expression sites of SPARC in inflammation and tumors. Understanding the structure of SPARC and the cause of its overexpression will help determine the pathogenesis of RA and facilitate the research on targeted therapy of late RA. In particular, the mechanism of SPARC as a potential target of RA and the latest research progress of functional nanomaterials to manipulate SPARC to treat inflammation and tumors were discussed.

## Discovery, function, and expression of SPARC

### Discovery and structure of SPARC

**FIGURE 1:**
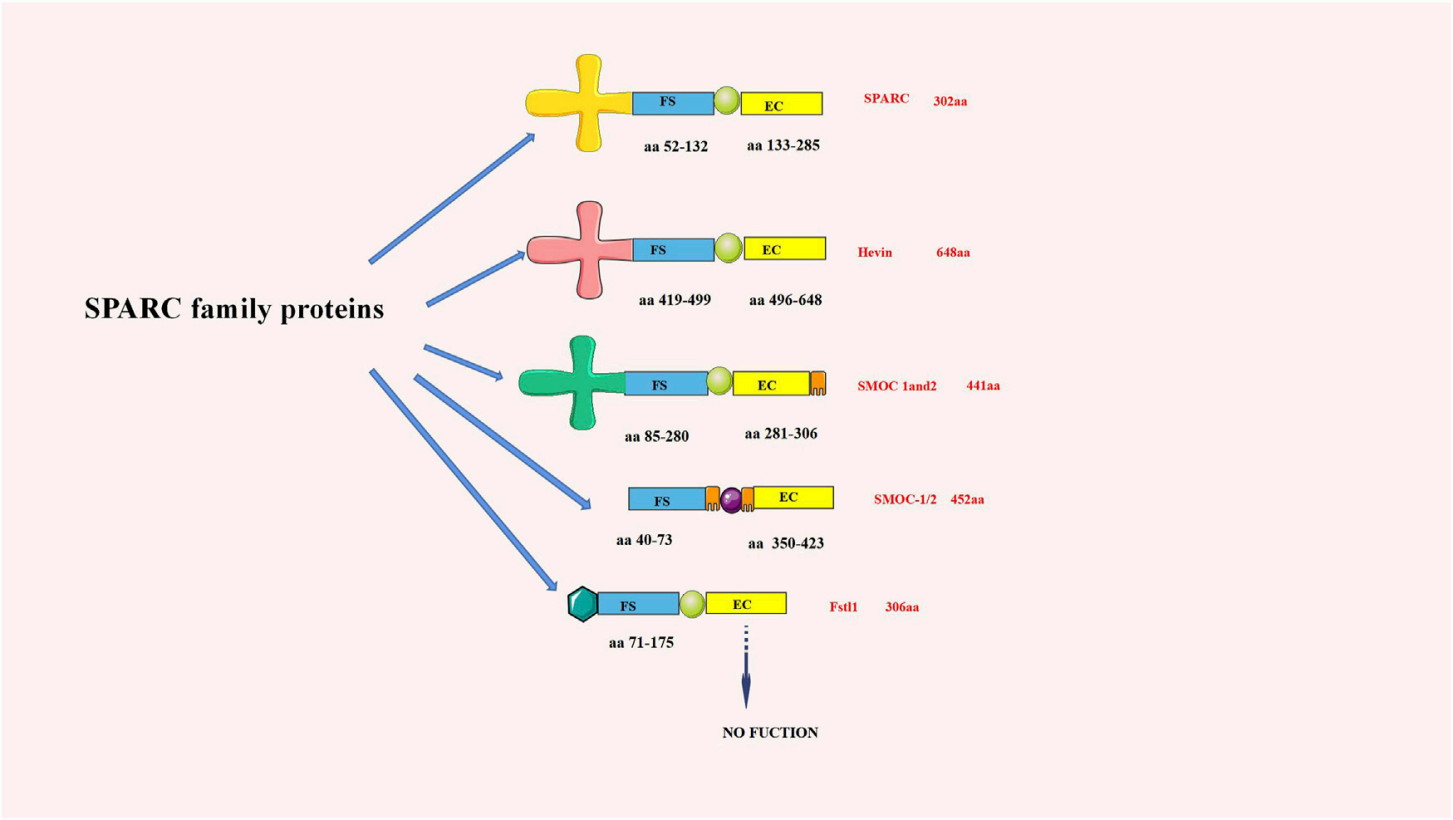
chematic diagram of the SPARC family structure (aa is the number of amino acids). SPARC is one of the most representative proteins in the SPARC family.

Termine JD first discovered that SPARC existed in the non-collagenous fetal bovine bone, which was the main component of its protein extract (Bradshaw, 2012). As an important part of the SPARC protein family, its domain comprises a cysteine-rich follistatin-like (FS) domain, an acidic N-terminal domain, and an *a*-helical extracellular (EC) calcium-binding domain with an EF-hand motif. Due to its structural properties, SPARC can bind various extracellular components (Gaudet et al., 2011). SPARC, equally known as BM-40 or 43K protein, has two calcium-binding sites on the protein, the N-terminal acidic domain, which binds 5 to 8 Ca^2+^ with less affinity, and the EF-hand motif located in the C-terminal domain, which binds Ca^2+^ ions with high affinity (Termine et al., 1981). The SPARC structure and SPARC protein family are shown in Figure 1.

### Physiological function of SPARC

SPARC is overexpressed during tissue renewal and repair. As a repair protein, SPARC plays an important role in manipulating cell proliferation, migration, and cytokine expression (Ng et al., 2013). Susceptibility of SKM-1 cells to Ara-C is enhanced with elevated levels of SPARC expression, accompanied by accelerated cell cycle restriction and apoptosis (Liang et al., 2022). Knockdown of the SPARC gene prolongs fibrosis during wound healing (Luo et al., 2019). Fibrotic changes *in vitro* and *in vivo* are effectively eliminated by specific suppression of SPARC (Wang et al., 2010). SPARC curbs apoptosis and inhibits the aggression and diversion of ovarian cancer cells (Chen et al., 2012). SPARC takes the center stage as ovarian cancer grows. The tumor suppressor gene, SPARC gene, is underexpressed in gastric cancer cell family (Zhou, 2018). SPARC inhibits the apoptosis of bone marrow stromal cells by inhibiting their proliferation (Deng, 2018). The activity of patients with ankylosing spondylitis can be assessed by detecting peripheral blood mononuclear cells and serum SPARC levels (Wang and Yang, 2018). As a matrix cell protein, SPARC is widely distributed in the eye and has the function of communicating cell and extracellular matrix signal transmission (Xiang and Tang, 2018). Silencing the SPARC gene reduces high fluorine-mediated cytotoxicity and inhibits apoptosis (Zhao et al., 2019). The degree of the expression level of SPARC protein can improve insulin resistance of adipocytes, thereby improving the insulin sensitivity of GK rats (Yao et al., 2020). As a stromal cell glycoprotein, SPARC is overexpressed in most inflammatory sites. SPARC has an irreplaceable role to play in prognosis and cure for cancer by regulating the proliferation, migration, and apoptosis of tumor cells. Knockdown or elimination of the SPARC gene can affect the speed of wound healing and reduce the effects due to cytotoxicity. SPARC protein can stimulate skin tissue fibrosis and increase fibroblast proliferation and collagen deposition.

### Expression and regulation of SPARC

**FIGURE 2:**
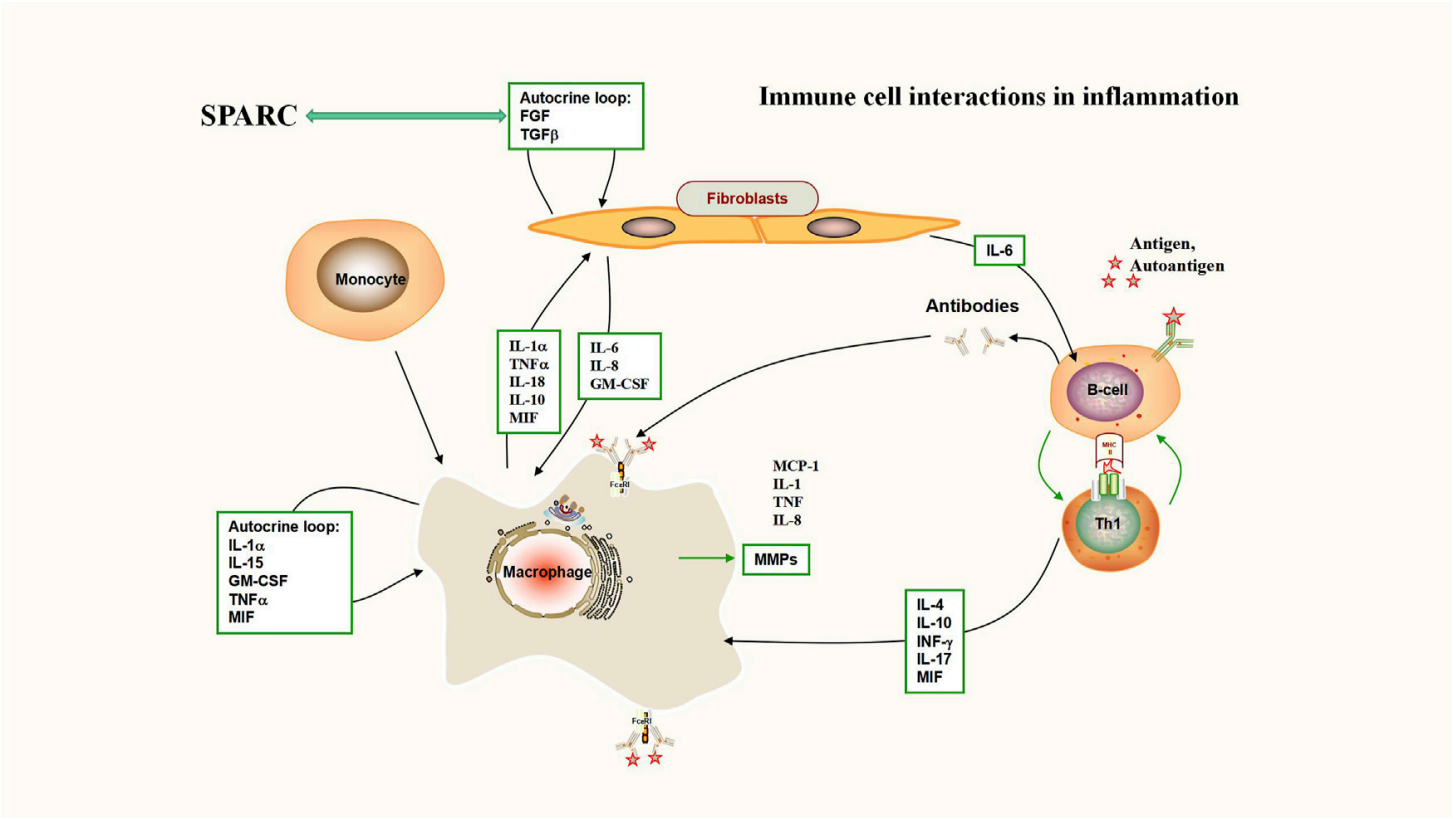
Immune cell interactions in inflammation (interaction between SPARC and TGF-β). Deficiency of TGF-β signaling effectively protects against inflammatory joint erosion in the arthritis model. Interaction between SPARC and TGF-β plays a key role in inflammatory sites including rheumatoid arthritis.

The expression of SPARC was significantly elevated during embryonic development as compared to normal adult tissues (Wong and Sukkar, 2017). SPARC expression levels are highly increased in epithelial cells during tissue injury, inflammation, and abnormal growth, such as tumors (Chiodoni et al., 2010). For some highly metastatic tumors, SPARC expression shows a high expression status, such as glioblastoma (Feng and Tang, 2014). SPARC, a potential indicator of inflammation and interferon responses, has the ability to shift anti-inflammatory macrophages into pro-inflammatory macrophages (Ryu et al., 2022). Huge expression levels of SPARC are usually detected at the sites of inflammation (Maltzman, 2022). Compared to normal cartilage, there are a large number of SPARC in the upper and middle areas of arthritic cartilage in RA patients, which can promote the decomposition of the ECM that is located on the surface area of arthritic cartilage through matrix metalloproteinases (MMPs). The proliferation of chondrocytes in the middle and deep layers can be regulated by SPARC. The unusual synthesis and degradation of SPARC might be ascribed to the destruction of the cartilage and bone (Li et al., 2023). Suppression of SPARC gene expression could inhibit the proliferation of human keloid fibroblasts (HKFs), block the process of the cell cycle, and promote apoptosis, and the downregulation of the TGF-β signaling pathway may have some relevance to this mechanism (Li, 2022). As an important factor, SPARC participates in the regulation of multiple signaling pathways, such as JAK/STAT and mTOR (Wang et al., 2019). SPARC regulates osteoblast mineralization through the p38 MAPK pathway (Zhu, 2020). Expression levels of SPARC and SPARCL-1 are substantially elevated in Glu-induced hippocampal neuronal injury (Agostina et al., 2021), and SPARC and SPARCL-1 regulated autophagy through the AKT–mTOR pathway to affect Glu-induced hippocampal neuron injury (Chen, 2020). SPARC is involved in VEGF-induced fibrosis in HTF cells, and it will be a new therapeutic opportunity for antifibrotic strategies after the completion of filtration surgery (Xiang and Tang, 2018). SPARC promotes the apoptosis of HUASMCs through the mitochondrial pathway, inhibits the proliferation rate of HUASMCs, thereby reducing their cell viability, promotes the secretion of gelatinase (MMP2 and MMP9), and leads to the degeneration of the extracellular matrix and internal elastic layer in the vessel wall (Ye, 2017). The expression and regulation mechanism of SPARC are shown in Figure 2. The following section introduces the latest application and research progress of SPARC as an underlying and efficient curative target for inflammatory diseases and tumors as well as functional nanomaterials to manipulate SPARC to treat diseases.

## Rheumatoid arthritis

Rheumatoid arthritis (RA) is an autoimmune disease that occurs along with a phenomenon that influences both joints (Ainsworth et al., 2022). The typical pathology of RA is characterized by changes in inflammatory cells and deformation of small joints. Patients with severe RA have significant atherosclerosis (Wang Z. et al., 2022). Macrophages are key cells in the treatment of RA. Macrophage activation is the main feature (Xu Y. et al., 2022). SPARC acts as a multifunctional modulator of parenchymal cells with enhanced expression in smooth muscle cells and macrophages in the atherosclerotic lesion stage (Raines et al., 1992). The high representation level of SPARC in macrophages can be an effective way to treat RA. Macrophages and FLS became the main target cells for targeted therapy of RA (Tu et al., 2022). SPARC may play a role in arthritis by stimulating MMP synthesis in synovial fibroblasts (Tremble et al., 1993). Multiple research experiments have found that synovial inflammation in arthritic mouse models can significantly be inhibited by blocking the NF-κB pathway. Due to the increased amount of TGF-β expression in RA-FLS, it clearly promotes the destruction and erosion of cartilage and bones by FLS (Stanford et al., 2016). SPARC exhibits anti-inflammatory effects through negative regulatory mechanisms on TGF-β and NF-κB signaling (Puolakkainen et al., 2003). SPARC could be a promoter of ECM degradation through the action of MMPs.

### Expression of SPARC in RA

Persistent synovitis and systemic inflammation are key features of RA (Giachi et al., 2022). A UK study found that the lowest prevalence of RA was much higher for women than that for men (Charles et al., 2013). There have been reports that the SPARC expression level in the ground and middle layer of articular cartilage in RA patients is significantly increased, and the level of synovial fluid and synoviocytes is increased (Nakamura et al., 1996). The discovery of SPARC provides new insights into it as a new target. IHC verified the high expression of SPARC in RA inflammatory joints (Liu et al., 2019), but SPARC staining is absent in normal cartilage. Synoviocytes from both RA and OA joints were found to have increased SPARC synthesis (Nakamura et al., 1996). At present, there are few reports on the high expression of SPARC in the RA joint synovium. Finding effective ways to treat RA is a major medical challenge. The discovery of SPARC, a potential target and therapeutic index, is expected to help advance the effective treatment of RA.

### Regulatory mechanism of SPARC in RA

**FIGURE 3:**
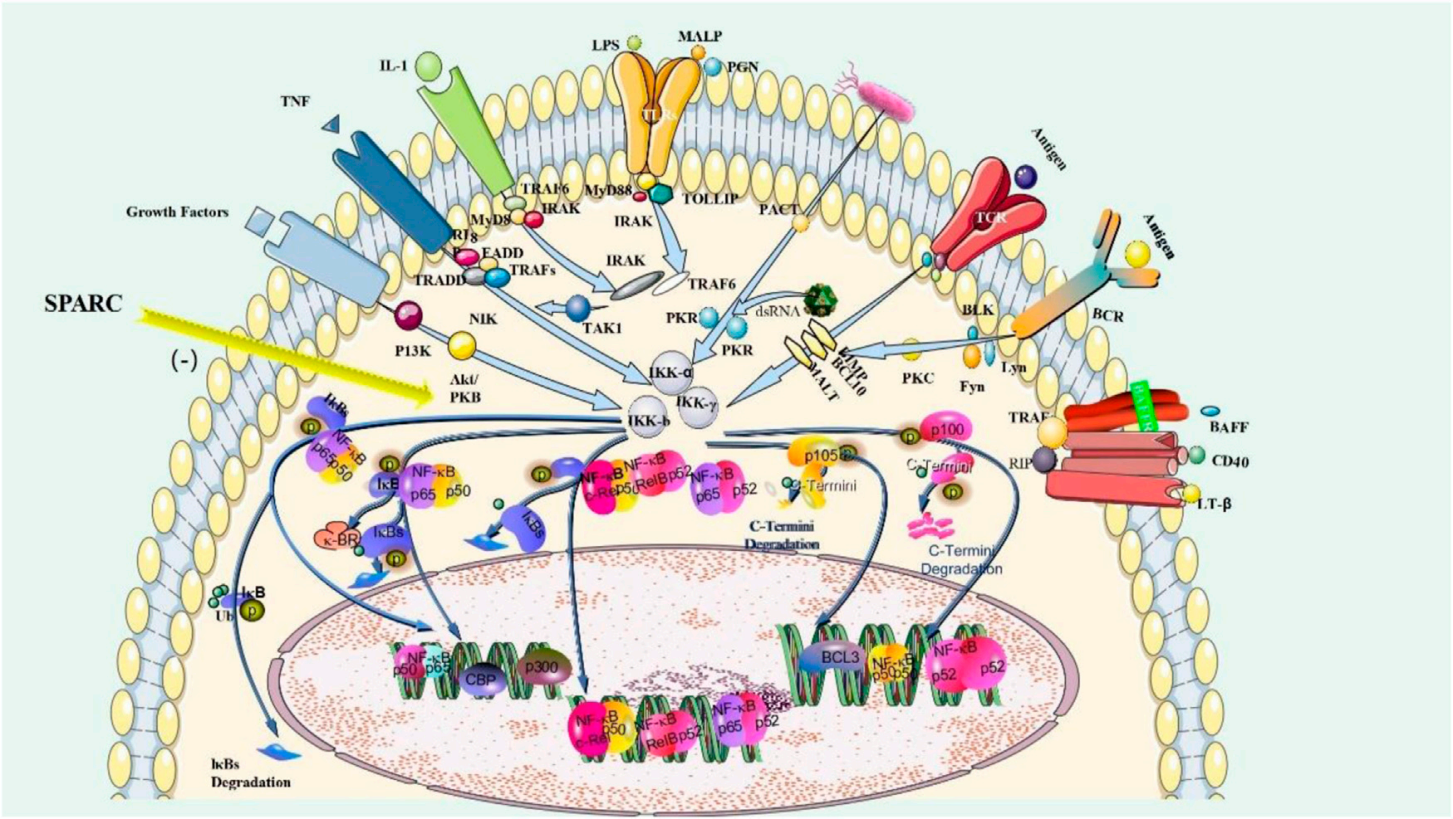
Negative regulation mechanism of SPARC on NF-κB. SPARC inhibits the proliferation of macrophages and HFLS-RA by negatively regulating the NF-κB pathway and promotes their apoptosis by playing an anti-inflammatory role.

SPARC, as a matrix protein, participates in the remodeling of normal and abnormal tissues (Puolakkainen et al., 2003). SPARC exhibits anti-inflammatory effects through an adverse regulator of TGF-β and NF-κB signaling (Sangaletti et al., 2011). The negative regulation mechanism of SPARC on NF-κB is shown in Figure 3. Previous studies have shown that the concentration of TGF-β that is required for the maximal stimulation of SPARC synthesis is basically consistent with the level of TGF-β in RA joints (Fava et al., 1989). SPARC can regulate the apoptosis of immune cells and limit the apoptosis of B-cell precursors (Tripodo et al., 2012). By culturing rabbit articular chondrocytes, it was found that TGF-β significantly increased SPARC levels, whereas IL-1β significantly decreased SPARC (Nakamura et al., 1996). Modulation of inflammatory cells and cytokines is an important therapeutic target to control inflammation in RA. As an important factor, TGF-β has become a hotspot in the study of RA treatment (Cheng et al., 2022). The interplay of SPARC with TGF-β is shown in Figure 3. The level/activity of TGF-β in patients with RA was found to be markedly higher than that in healthy individuals. In a model of inflammatory arthritis, the deficiency of TGF-β signaling effectively prevents inflammatory joint erosion (Xia et al., 2022). Inhibiting the NF-𝜅B pathway can inhibit RA-FLS and RAW 264.7 cell proliferation, induce apoptosis, and improve RA inflammation (Yang Y. P. et al., 2022). For example, aspirin promotes apoptosis in HFLS by downregulating the NF-κB pathway (Zhang X. et al., 2018). Inflammation is an inescapable pathological feature of RA. A central regulatory role in the pathological process of RA is played by the activation of the NF-κB signaling path (Xu Y. J. et al., 2022). Pathological NF-κB activation assumes a significant task at the time of emergence and development of rheumatoid arthritis (Zhou and Zhang, 2012). SPARC is shown at high levels in RA joints and has regulatory effects on TGF-β and NF-κB signaling paths. At present, the research on the mechanism of RA is gradually deepening, and the research on TGF-β and NF-κB signaling pathways in RA is increasing. SPARC, as a matrix protein, plays an anti-inflammatory role through the negative regulatory mechanism of TGF-β and NF-κB pathways. Massive proliferation of HFLS is the basic pathological feature of RA. The character of SPARC in the promotion of HFLS-RA apoptosis is bound to provide an opportunity for SPARC to become a fresh target for the cure of RA. The regulation mechanism of SPARC in RA is shown in Figure 3.

### Role of SPARC in RA

**FIGURE 4:**
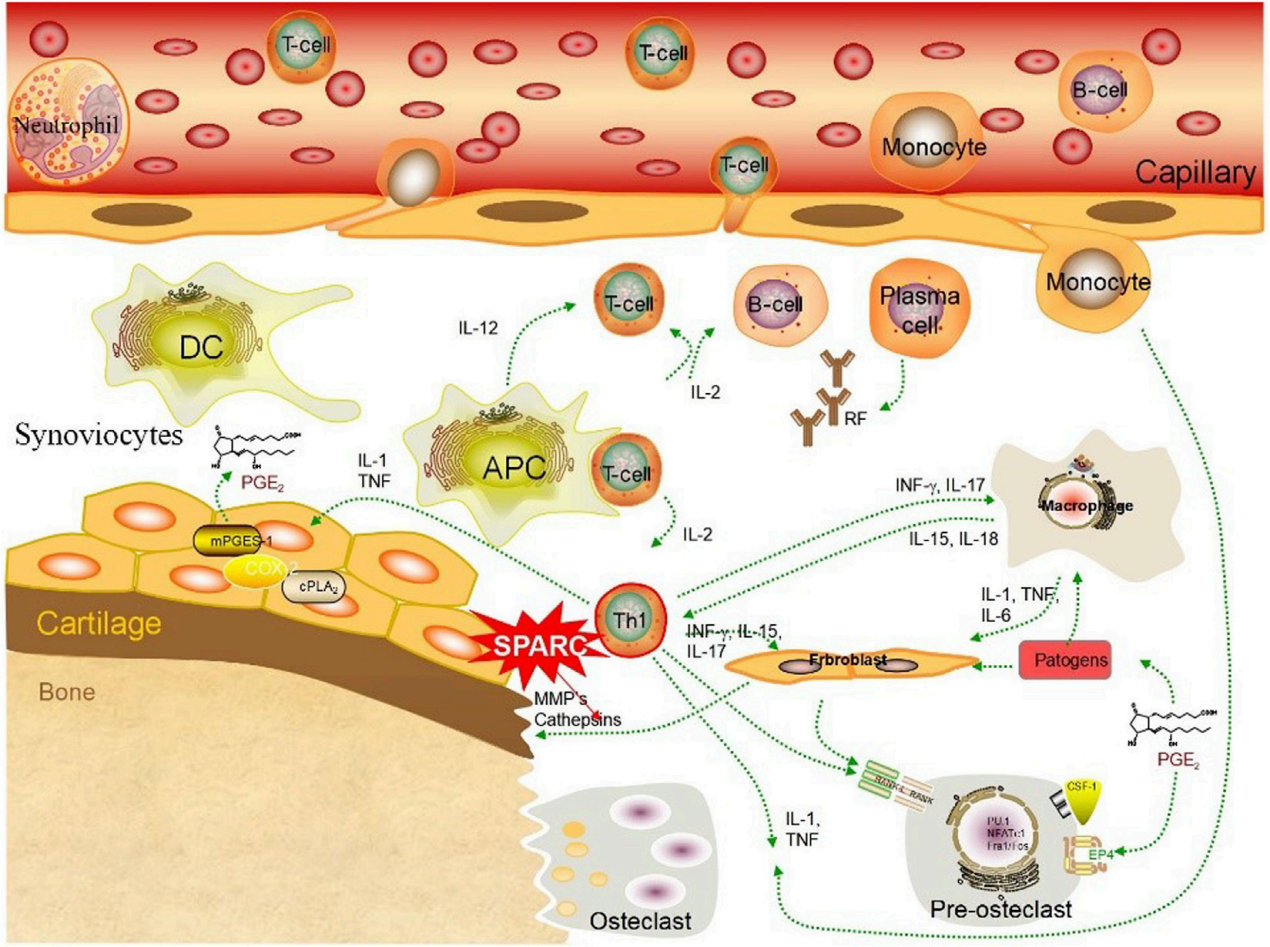
SPARC promotes ECM degradation through MMPs. SPARC promotes the apoptosis of HUASMCs through the mitochondrial pathway, inhibits the proliferation rate of HUASMCs cells, thereby reducing their cell viability, promotes the secretion of gelatinase (MMP2 and MMP9), and leads to the degeneration of the extracellular matrix and internal elastic layer in the vessel wall

SPARC has a dual role in resorbing and regenerating arthritic cartilage. Numerous studies have demonstrated synovial tissue heterogeneity in RA joints (Ouboussad et al., 2019). HFLS forms the synovial intima, which has an important function in the destruction of RA joints (Firestein, 2004). FLS presents an aggressive phenotype in joint destruction and inflammation in patients with RA (Nygaard and Firestein, 2020). FLS-mediated excessive production of MMPs damages the collagen-rich structure of the joint tissue at the pannus–cartilage interface of rheumatoid joints and promotes FLS invasion (Nygaard and Firestein, 2020). MMPs play a key role in the remodeling of the ECM (Zhang et al., 2021). SPARC might promote the decomposition of the ECM through the action of MMPs on the surface area of arthritic cartilage (Tremble et al., 1993). SPARC promotes ECM degradation through MMPs, as shown in Figure 4. Irregular synthesis and degradation of SPARC may be associated with cartilage and bone deterioration. SPARC exudes a range of bioactive peptides containing the (K)GHK array that can affect angiogenesis and synovial hyperplasia to influence vascular remodeling (Lane et al., 1994). The pannus phenomenon is closely related to SPARC. FLS-RA is the main cell in RA joint destruction, and the excessive production of MMPs mediated by it will destroy the joint tissue structure.

As a chronic autoimmune disease caused by immune system dysfunction, RA is very important to find an active therapeutic marker for the growth of RA. As a hidden target of RA, SPARC can have excellent benign effects on RA joints based on the negative regulation of TGF-β and NF-κB pathways. Currently, the focus of RA curative research is on indicators such as MMPs and receptor activation of the NF-κB ligand (RANKL) (Meng et al., 2022). As an overexpressed substance in RA articular cartilage, SPARC participates not only in the ECM degradation process promoted by MMPs but also in negatively regulating TGF-β and NF-κB pathways. Therefore, SPARC becomes a dual possible target for the therapeutic cure of RA. In the pathogenesis of RA, SPARC can exhibit its full anti-inflammatory effect. The discovery of SPARC, a potential target, has significant implications for treating and prognosticating RA in the years to come.

## Tumors

The microenvironment of the tumor is a vital regulator of tumor initiation, progression, and migration. SPARC is covered in interactions between the tumor and stroma and influences cancer growth by influencing tumor invasion (Camacho et al., 2020). High level of SPARC expression is typically linked to aggressive and highly metastatic tumors. Based on this feature, SPARC is often used as a prognostic marker for different cancers. Interestingly, further clinical exploration found that SPARC could be used as a candidate aim for the control of cancer (Podhajcer et al., 2008). SPARC is categorized as a promising target and indicator for treating various cancers.

### Gastric cancer

Risk factors for stomach cancer, which is one of the extremely common and high-mortality cancers, include dietary habits and bacterial infections (Smyth et al., 2020). Gastric cancer affects dietary life for a long time for patients, bringing great changes and troubles. Discovering new potential therapeutic or prognostic targets will bring good news to patients with gastric cancer.

#### Regulatory mechanisms of SPARC in gastric cancer

Through network pharmacology analysis, SPARC expression has been shown to be strongly correlated with the bad prognosis of GC. SPARC can be used as a marker of its bad prognosis (Li et al., 2019). Subsequently, clinical studies have found that SPARC is a possible marker for diagnosing and prognosticating gastric cancer and an important factor regulating various signaling pathways (Wang et al., 2019). The SPARC gene acts to suppress epigenetic tumorigenesis (Shao, 2018). SPARC brings new targets and therapies to treat gastric cancer (Zhou, 2018). SPARC may support the invasion, metastasis, and angiogenesis of gastric cancer cells (Ma Y. S. et al., 2017). Genome-scale analysis reveals that SPARC is a marker for the diagnosis and prognosis of gastric cancer (Xu et al., 2018). A meta-analysis was performed to elucidate that SPARC was a prospective clinical predictor of survival in gastric cancer patients (Wang et al., 2014). The impact of SPARC on the proliferation and migration of gastric cancer cells was induced by regulating developmental factor arrival (Phan et al., 2007). SPARC levels are inversely correlated with GC clinicopathological factors (Zhang et al., 2014). Clinical data showed that the proficiency of SPARC was significantly reduced in a group of patients with gastric cancer after treatment (Gao et al., 2015). Early network pharmacology provided data support for the discovery of SPARC, a new prognostic indicator, and later, a clinical research further confirmed this point of view.

#### Role of SPARC in gastric cancer

The investigators revealed that the level of SPARC in GC may be related to the induction of GC cells (Li Z. et al., 2016). SPARC positivity in gastric cancer tissue was higher than in non-cancerous gastric tissue (Gao et al., 2017). SPARC and VEGF jointly promote angiogenesis in tumor sites (Wang et al., 2012). Human gastric cancer cell invasion and growth may be inhibited by the downregulation of SPARC (Yin et al., 2010). In tumor tissues lacking SPARC, the tumor growth rate and volume were significantly reduced (Ma et al., 2021). SPARC was upregulated in gastric tumor samples and had a strong relationship with poorer overall survival. The prognostic significance of risk score models for gastric cancer prediction can be refined by analyzing differentially expressed genes (DEGs) using SPARC (Shan et al., 2021). The PCR method reveals the important role of SPARC epistasis in gastric carcinogenesis (Chen et al., 2018). Downregulating the expression of VGEF can block the angiogenesis of gastric cancer (Zhang J. L. et al., 2012). Increased levels of SPARC expression are closely related to poor outcomes in GC in Kaplan–Meier survival analysis (Liao et al., 2018). The level of expression of SPARC is strongly correlative to the level of GC cells (Zhu et al., 2015). SPARC may participate in gastric cancer by affecting the tumor microenvironment transfer (Mo et al., 2017). As a target gene for treating GC, SPARC has the exclusive function of detecting drug sensitivity (Zhao et al., 2021). The discovery of SPARC will lead the way for further research on the role of circRNA in the GC body (Tian et al., 2020). SPARC is a resource for conducting a survey on the potential biomarkers and targets for the diagnosis, prognosis, and treatment of GC (Hao et al., 2019). SPARC is a key growth factor in gastric cancer (Zhang J. L. et al., 2012). SGC7901 cells expressing high levels of SPARC are more active and resistant to anticancer drugs than MGC803 cells expressing low levels of SPARC (Li A. et al., 2015). The period for the greatest disease response in cancer patients is generally in the sophisticated stage of cancer. The discovery of a novel predictor is essential for gastric cancer patients. In patients with stage II/III gastric cancer, high levels of SPARC gene expression may be a valuable prognostic factor (Suzuki et al., 2018). The Gene Expression Omnibus (GEO) network database found that SPARC overexpression levels were linked to reduced overall GC survival rates (Yan et al., 2018). SPARC is located in peritumoral fibroblasts and rarely observed in cancer cells’ cytoplasm (Nakajima et al., 2018). Moving forward, the clinical detection of the prognosis of GC patients can be more targeted at HFLS around CG cells. SPARC, as a prognostic indicator, will bring convenience to the detection of GC prognosis.

Network pharmacology brings data support for SPARC as a GC prognostic indicator, and clinical pharmacology experiments such as WB continue to verify that SPARC and GC are inseparable. As an effective prognostic indicator for GC, the high expression of SPARC was accompanied by the rapid growth of GC cells.

### Hepatocellular carcinoma

One of the most lethal cancers worldwide is hepatocellular carcinoma (HCC) (Piñero et al., 2020). As the sixth most common cancer worldwide, the overexpression of SPARC reduces HCC proliferation during spheroid cell culture. SPARC improves patient survival by inhibiting the tumorigenic ability of host macrophages. Additionally, SPARC leads to its reduced tumorigenicity by inducing mesenchymal–epithelial transition (MET) (Atorrasagasti et al., 2010). The dual regulatory effect of SPARC on HCC has become the biggest breakthrough in the targeted therapy of HCC.

#### Regulatory mechanism of SPARC in hepatocellular carcinoma

When HCC patients develop disease, the mRNA of SPARC is expressed at a high standard *in vivo* (Gao et al., 2021). The overexpression of SPARC was shown to strengthen the cytotoxic effect of sorafenib in Hep3B and HepG2 cells, significantly elicited LDH release, and induced oxidative stress compared with parental cells (Hua et al., 2021).

#### Role of SPARC in hepatocellular carcinoma

A study found that HCC cells in SPARC knockout mice were significantly affected (A et al., 2021). As the mechanism of SPARC inactivation in HCC, SPARC methylation becomes a biological indicator of HCC prognosis (Zhang Y. et al., 2012). A meta-analysis was conducted and revealed that an elevated SPARC level was correlative with the poor survival of HCC (Yang X. et al., 2022). SPARC affects HCC cell viability through the regulation of the ERK1/2-MMP2/9 pathway (Liu et al., 2020). The growth rate and angiogenesis of HCC are tightly correlated with the high expression level of SPARC (Lau et al., 2006). MuiR-29a-overexpressing HCC cells inhibited phosphorylation of AKT/mTOR downturn of SPARC (Zhu et al., 2012). The identification of this new target of SPARC has made a historic leap forward in the targeted care of HCC (Hua et al., 2015). SPARC is extremely expressive in severe HCC tissue sections without abnormalities found in normal tissues (Le Bail et al., 1999). SPARC has been confirmed to be a *bona fide* target of miR-211 (Deng et al., 2016). Sorafenib is the first-line drug to treat advanced HCC, and SPARC is considered a potential drug target (Goyal et al., 2019). The fluorescent probe method detected that SPARC was significantly higher in HCC patients than in healthy individuals (Segat et al., 2009). Increased tumor volume of HCC in nude mice is supported by the overexpression of SPARC (Jiang et al., 2019). SPARC was also a central gene with significant diagnostic value (Ye et al., 2020). The follistatin-like domain of the SPARC protein decreased the level of E-cadherin expression (Sun et al., 2018). SPARC has the driving force to regulate the progression of HCC (Wan et al., 2015). SPARC has been entangled in the progress of fibrosis in the liver (Nakatani et al., 2002). Based on the lack of effective therapeutic targets for HCC, SPARC will play a prominent role as a new target (Ang et al., 2016).

SPARC will be a potent target for the management of HCC. Looking ahead, strong and effective treatment can be achieved using functional nanomaterials by manipulating SPARC, reducing the pain of patients.

### Breast cancer

Breast cancer, the most prevalent cancer in females globally, is characterized by bone metastases. SPARC has the ability to inhibit osteoclast activation in the microenvironment besides inhibiting breast cancer cell migration and invasion. SPARC can be a viable curative target for treating breast cancer metastasized to the bone (Ma J. et al., 2017). Compared with tumor cells, SPARC appears at higher levels in the stromal cells of the tumor (Shi et al., 2022). After stage treatment, the expression of SPARC in the stromal cells will be used as a predictive pointer for breast cancer.

#### Regulatory mechanism of SPARC in breast cancer

Triple-negative breast cancer occurs in up to 20% of breast cancer families. In triple-negative breast cancer, SPARC is widely expressed (Lindner et al., 2015). The high affinity between nal-paclitaxel’s functional structural protein, HSA, and SPARC is responsible for its excellent efficacy in the management of breast cancer (Yardley, 2013). There is an urgent need for alternative therapeutic strategies in TNBC on the basis of tumor-specific molecular targets. Studies have demonstrated a novel crosstalk between proteases and stromal cellular proteins in the tumor microenvironment through the limited proteolysis of SPARC and have found that SPARC may serve as a possible therapeutic target in TNBC (Alcaraz et al., 2021). SPARC expression in CAF is an exclusive prognostic factor for poor prognosis in TNBC (Alcaraz et al., 2022). Breast cancer afflicting women must be addressed well. The emergence of a good target and prognostic indicator, SPARC, is going to open the door to a whole new world in the treatment of breast cancer.

#### Role of SPARC in breast cancer

Breast cancer tissues and most breast cancer cells have a higher level of SPARC (Li X. L. et al., 2022). A high SPARC level correlated with breast cancer cell differentiation becomes a new therapeutic approach (Bawazeer et al., 2018). SPARC is downregulated during breast cancer development (Nagai et al., 2011). As a 32 kDa secreted glycoprotein, the elevated level of SPARC was related to the overall survival of patients (Watkins et al., 2005). SPARC acts as a prognostic indicator. After chemotherapy, the overall survival of patients with senior SPARC expression was markedly shortened (Zhu et al., 2016). Network pharmacology found that SPARC can be used as the basis for early breast cancer diagnosis (Azim et al., 2013). SPARC is an inhibitor of the migration and invasion of breast cancer, while resisting the platelet deficiency caused by it (Koblinski et al., 2005). Breast cancer progression to the S phase was slowed down by SPARC (Dhanesuan et al., 2002). SPARC might be a helpful marker for aggressive, metastatic-prone tumors (Wong et al., 2008). SPARC is able to induce the activation of MMP-2 in breast cancer cell lines (Gilles et al., 1998). Appealing targets for anti-metastatic therapy in breast cancer are SPARC and its secondary effectors (Güttlein et al., 2017). SPARC is accompanied by a reduced extracellular matrix (Hellinger et al., 2020). SPARC was found to be 69.3% positive in cancerous tissues (Guo et al., 2017). SPARC upregulation is a system whereby PTEN controls the deposition of collagen in the mammary stroma (Jones et al., 2021). SPARC-mediated degradation of the ECM and its potential link to MMPs could lead to breast cancer development (Kim et al., 2017). Compared to those with poor SPARC expressions, those with positive SPARC expressions had a 2.34-fold increased risk of death (Hsiao et al., 2010). Plasma concentration of SPARC was less in normal females, suggesting an anti-adhesive effect of circulating SPARC (Maroni et al., 2015). A high expression of bone remodeling protein-SPARC in a highly malignant phenotype can be observed (Lukianova et al., 2022). Screening of aggressive cancer-associated DEGs and important pathways revealed that SPARC could be a promising target gene in metastatic breast cancer (Chen et al., 2015). The upregulation of SPARC was observed after neoadjuvant chemotherapy is correlated with chemotherapy resistance in breast cancer patients (Wang et al., 2016). A high expression of bone remodeling protein-SPARC in a highly malignant phenotype can be observed (Szynglarewicz et al., 2016). The inhibition of SPARC secretion by oleic acid ultimately favors T-cell activation as an underlying mechanism for the reduction of breast cancer aggressiveness (Bellenghi et al., 2022). The vast majority of stromal cells showed abundant cytoplasmic SPARC reactivity (Barth et al., 2005). Patients with a high level of SPARC have increased survival rate (Beck et al., 2008).

Triple-negative breast cancer has been plagued clinically due to the absence of a suitable target. SPARC, as a target protein that inhibits the cell cycle of breast cancer, is bound to increase the target of chemotherapy medicines. The combination of functional nanomaterials and SPARC will bring good news to patients in the future.

### Non-small-cell lung cancer

Investigations have shown that SPARC promotes pathological reactions in non-small-cell lung cancer (NSCLC) and idiopathic pulmonary fibrosis by encouraging microvascular remodeling and overproduction of ECM proteins (Wong and Sukkar, 2017). The primary reason of cancer death worldwide is NSCLC Mirhadi et al., 2022). SPARC was a latent prognostic biomarker in NSCLC (Fabrizio et al., 2020).

#### Regulatory mechanism of SPARC in NSCLC

Lung cancer growth may be inhibited by the elevated level of SPARC mRNA in lung cancer tissues (Wang B. et al., 2018). Network pharmacology analysis shows that SPARC is expressed at a highly increased level in NSCLC tissues (Ma G. Y. et al., 2022). SPARC is a biologically relevant mechanism that is upregulated in the development of lung cancer as a specific site that regulates collagen binding (Kehlet et al., 2018). SPARC overexpression in A549 and H1299 cells drives migration and EMT, respectively (Sun et al., 2018). The downregulation of SPARC expression in H322 and A549 cells leads to the inhibition of cell invasion (Zhou et al., 2010). SPARC has the advantage of promoting, invading, and migrating lung cancer cells, making it a novel therapeutic target.

#### Role of SPARC in NSCLC

In two NSCLC cell lines, CL1-5 and H1299, SPARC treatment increased cell growth, migration, and the mesenchymal phenotype (Hung et al., 2017). SPARC can be a prognostic overall survival indicator (Huang et al., 2012). The upregulation of SPARC helps define the pathogenesis of NSCLC (Grant et al., 2014). Plasma of lung cancer patients contains overexpressed SPARC (Andriani et al., 2018). SPARC promotes growth and inhibits the apoptosis of lung cancer cells (Xu et al., 2019). SPARC produced by stromal cells supports a high degree of vascular maturation (Koukourakis et al., 2003). The positive representation rate of SPARC in stages I and II lung cancer was significantly lower than that in stages III and IV lung cancer (Zhang Z. et al., 2012). SPARC expression was upregulated in lung cancer cells via promoter demethylation and is correlative with a decreased DNA methyltransferase (DNMT) activity (Pan et al., 2008). SPARC increased the permeability of macromolecules through the integrin/focal adhesion/cell-promoted persistent activation of the alveolar epithelial junction axis (Conforti et al., 2020). The SPARC gene is a tumor-promoting gene (Yang et al., 2019). The SPARC gene is also a prognostic gene (Zhong et al., 2022). SPARC was significantly downregulated in hypermethylated tumors (Ito et al., 2005).

Network pharmacology and experimental studies have continuously confirmed the strong presence of SPARC in lung cancer. As a novel therapeutic target, the high-level expression of SPARC is mediated by promoter demethylation. Clinical cancer samples confirmed that SPARC can be a candidate therapeutic marker and prognostic indicator for the diagnosis of NSCLC.

### Melanoma

Melanoma significantly affects the work and study of patients. The emergence of novel targets and prognostic indicators is critical for the entire therapeutic community.

#### Regulatory mechanism of SPARC in melanoma

Cell migration, adhesion, cytoskeletal features, and cell shape are influenced by the inhibition of SPARC expression in human melanoma cells (Salvatierra et al., 2015). Fibroblast-like morphology in normal human melanocytes overexpressed SPARC (Haber et al., 2008). SPARC stimulates cathepsin B-mediated melanoma invasion through this mechanism using collagen I and α2β1 integrin as mediators (Girotti et al., 2011). SPARC can enhance the blocking effect of Pf4 on the phosphorylation of ERK and the metastasis of melanoma cells, and the synergistic effect of SPARC provides the possibility of targeting the secretome of cancer cells for therapeutic development (Zeng et al., 2021). SPARC is bound to become a novel target for the development of melanoma therapies.

#### Role of SPARC in melanoma

The growth of melanoma is not regulated by exogenous SPARC or by stromal tissue, but solely by the amount of SPARC that is yield by the malignant cells themselves (Prada et al., 2007). SPARC could become a new target to stop the progression of melanoma (Maloney et al., 2009). SPARC limits p53 levels by activating Akt and MDM2, and gaining SPARC expression in the development of melanoma confers a survival advantage by the inhibition of p53-dependent apoptotic signaling pathways (Fenouille et al., 2011). SPARC maintains a high-level expression in melanoma (Fenouille et al., 2012). Interactions between melanoma and other cells are facilitated in a SPARC-dependent form (Defresne et al., 2011). Normal melanocytes do not express SPARC (Ledda et al., 1997). SPARC is a possible leader for the treatment of melanoma (An et al., 2013). SPARC is a stromal cell protein associated with melanoma invasiveness (Rocco et al., 2011). SPARC overexpression enhances vascular extravasation (Tichet et al., 2015). Serum levels of SPARC were significantly higher in individuals with melanoma than those in healthy donors (Ikuta et al., 2005). SPARC is found to be strongly expressed in paraffin-embedded samples of melanoma (Pieniazek et al., 2016). The proliferation of melanoma is associated with the regulation of the expression of proteins involved in the epithelial–mesenchymal transition and with the suppression of MMP-2 and activation of MMP-9 (Cerezo et al., 2013). SPARC plays an important role in the tumor evasion of immune surveillance by reducing anti-tumor PMN action (Alvarez et al., 2005). Invasive melanoma cells may invade intraepidermal regions and produce epidermal melanoma metastases (EMM) (Meling et al., 2021). SPARC aids cell expansion and could be a possible target for melanoma treatment (Horie et al., 2010). Desmoplastic melanoma (DM) is a unique form of melanoma in which SPARC expression is extremely high (Garrido et al., 2014). The SPARC gene is a key gene affecting the migration of melanoma (Kaochar et al., 2018). SPARC is one of the indicators that helps identify the progression of melanoma (Chakraborty et al., 2009). SPARC-enriched tumors had a substantially higher percentage of vascular occupancy (Ordonez et al., 2005). The utilization of therapeutic genes that are directed by the SPARC driver could be a useful approach for the treatment of cancer (Lopez et al., 2006). The high level of SPARC in acidic media is considered to be an essential potential for the invasive activity of tumors (Kato et al., 2000). SPARC promotes metastasis and vascular extravasation in melanoma.

The high expression of SPARC was found by conducting a study on melanoma specimens. SPARC was found to be a key target for treating melanoma. SPARC can enhance the permeability of blood vessels, its high expression in acidic media provides motivation, and it is a leader for treating melanoma.

### Esophageal cancer

Due to its highly aggressive nature and poor survival rate, esophageal cancer is one of the most aggressive cancers worldwide (Domper Arnal et al., 2015). Esophageal cancer greatly afflicts patients and produces many complications.

#### Regulatory mechanism of SPARC in esophageal cancer

In the epithelial cells of esophageal cancer, SPARC is extremely expressed (Brabender et al., 2005). The expression of the SPARC gene is found to be increased in esophageal cancer (Luo et al., 2004). SPARC is highly shown in the specimen (Botelho et al., 2010). The protein expression pattern of SPARC is useful for developing reasonable strategies for precocious detection of high-risk features and for the prevention and management of ESCC (Xue et al., 2006). SPARC becomes a prominent target for esophageal cancer imaging (Zhao et al., 2022). SPARC is linked to unfavorable prognosis in patients with ESCC (Wu et al., 2017). SPARC has a strong correlation with MMP-2 expression, and this correlation could play a critical role in the growth of esophageal cancer (Yamashita et al., 2003). Laminin-5γ2 chain and SPARC might participate in esophageal SCC progression, and their concurrent expression is associated with adverse prognosis (Xue et al., 2011). Compared to control mucosa, SPARC is highly overexpressed in tumor tissues (Porte et al., 1998). The downregulation of SPARC may reduce cell movement and invasion involved in EMT via the p-FAK/p-ERK pathway, which could represent a novel therapeutic approach for ESCC (Zhang et al., 2020).

#### Role of SPARC in esophageal cancer

Peritumoral stromal cells (tumor-associated fibroblasts and macrophages) were the principal source of SPARC in the TME, and exogenous SPARC increased tumor cell invasion (Ma W. et al., 2022). SPARC is a cytoplasmic and nuclear protein found in ESCC cells (Che et al., 2006). A high SPARC expression in ESCC parenchyma with IHC is combined with lymph node metastasis and bad prognosis (Chen et al., 2017).

During the procedure of esophageal cancer, SPARC could be deployed as a potential medicinal target for endoscopic detection due to its extremely high expression in IHC. In areas with high incidence, early diagnosis is more important for esophageal cancer, which has resulted in the emergence of endoscopic techniques. The high expression of SPARC provides development potential and convenience for detection. Through literature search and analysis of clinical cancer samples, it is found that SPARC is located in the nucleus and cytoplasm of ESCC and SPARC can be considered as a prognostic indicator.

### Ovarian cancer

Ovarian cancer (OvCa) is currently the fifth most frequent cause of cancer-related mortality among females in the United States, and approximately 140,000 females globally die from ovarian cancer each year (Penny, 2020). SPARC has a therapeutic effect by inhibiting OvCa cell metabolism (Naczki et al., 2018).

#### Regulatory mechanism of SPARC in ovarian cancer

SPARC inhibits the NF-κB pathway mediating macrophage-induced ovarian cancer cell invasion (Said et al., 2008). SPARC suppresses the differentiation of ovarian cancer cells and transition of adipocytes to cancer (John et al., 2019). SPARC promoter methylation is an essential element in the occurrence and survival of ovarian cancer, and SPARC can be used as a therapeutic target and predictive marker for ovarian cancer (Socha et al., 2009). SPARC markedly reduced the proliferative, chemotactic, and invasive effects of LPA on ovarian cancer cell lines (Said N. A. et al., 2007). Knocking down SPARC production clearly reduced ovarian cancer cell proliferation, induced the apoptosis, and prevented the cells from invading and metastasizing. SPARC becomes a key factor in ovarian cancer development as it is overexpressed in aggressive subclones and ovarian cancer samples (Chen et al., 2012).

#### Role of SPARC in ovarian cancer

SPARC helps normalize the ovarian cancer malignant ascites microenvironment by downregulating the VEGF–integrin–MMP axis, reducing the levels and activation of bioactive lipids, and ameliorating downstream inflammatory (Said et al., 2007b). The expression of SPARC in ovarian cancer cells was negatively associated with the level of malignancy (Yiu et al., 2001). In the ovarian stroma containing malignant cells, particularly at the tumor–stroma interface in invasive tumors, elevated levels of SPARC mRNA and protein expression were found (Brown et al., 1999). SPARC has limitations on normal ovaries in premenopausal patients (Paley et al., 2000). SPARC may work as a tumor suppressor to reduce angiogenesis and lymphangiogenesis in ovarian cancer by decreasing the amount of VEGF-C and VEGF-D expression (Peng et al., 2017). SPARC modulates TGF-β1 fibrillar ECM deposition through a novel mechanism to influence cancer cell biology (Tumbarello et al., 2016). SPARC inhibits the αv- and β1-integrin-mediated attachment of ovarian cancer cells to the ECM (Said et al., 2007a).

Malignant ascites is the most common cause of death from ovarian cancer, and SPARC normalizes malignant ascites in the ovarian cancer microenvironment and improves inflammation through downregulating the VEGF–integrin–MMP axis. Due to the negative correlation among SPARC and ovarian cancer and the regulation of the microenvironment, SPARC could be a possible drug target for curing ovarian cancer.

### Other cancers

Neoadjuvant chemoradiotherapy can improve the tumor resection rate (Li Y. et al., 2016) and SPARC was found to be its central gene (Sun et al., 2020). Rectal cancer patients with high SPARC expression have poor prognosis. SPARC emerges as a prognostic indicator for patients with colorectal cancer undergoing chemoradiotherapy (Kurtul et al., 2017).

Bladder cancer (BCa) is the most prevalent malignancy of the urinary tract and remains one of the most frequent forms of cancer in the world (Dobruch and Oszczudłowski, 2021). The frequency and strength of SPARC expression negatively correlated with disease-specific survival in human bladder tumor tissues (Said et al., 2013). SPARC shows potential as a prognostic indicator of tumor recurrence or progression during prostate cancer theranostics but is affected by hematuria (Critselis et al., 2019). The level of the SPARC gene has a significant relationship with the histological type, pathological status, and prognosis of bladder cancer (Yamanaka et al., 2001). The SPARC level in human bladder cancer cell lines is negatively associated with the proliferation rate (Larson et al., 2010; Makridakis et al., 2010; Said, 2016).

Head and neck cancer is one of the most prevalent cancers globally (Siegel et al., 2017). In cDNA array analysis, the expression of SPARC in tumor regions was senior compared with adjacent normal regions. SPARC can enhance the proliferation and relocation of head and neck cancer cells (Chang et al., 2017).

**FIGURE 5:**
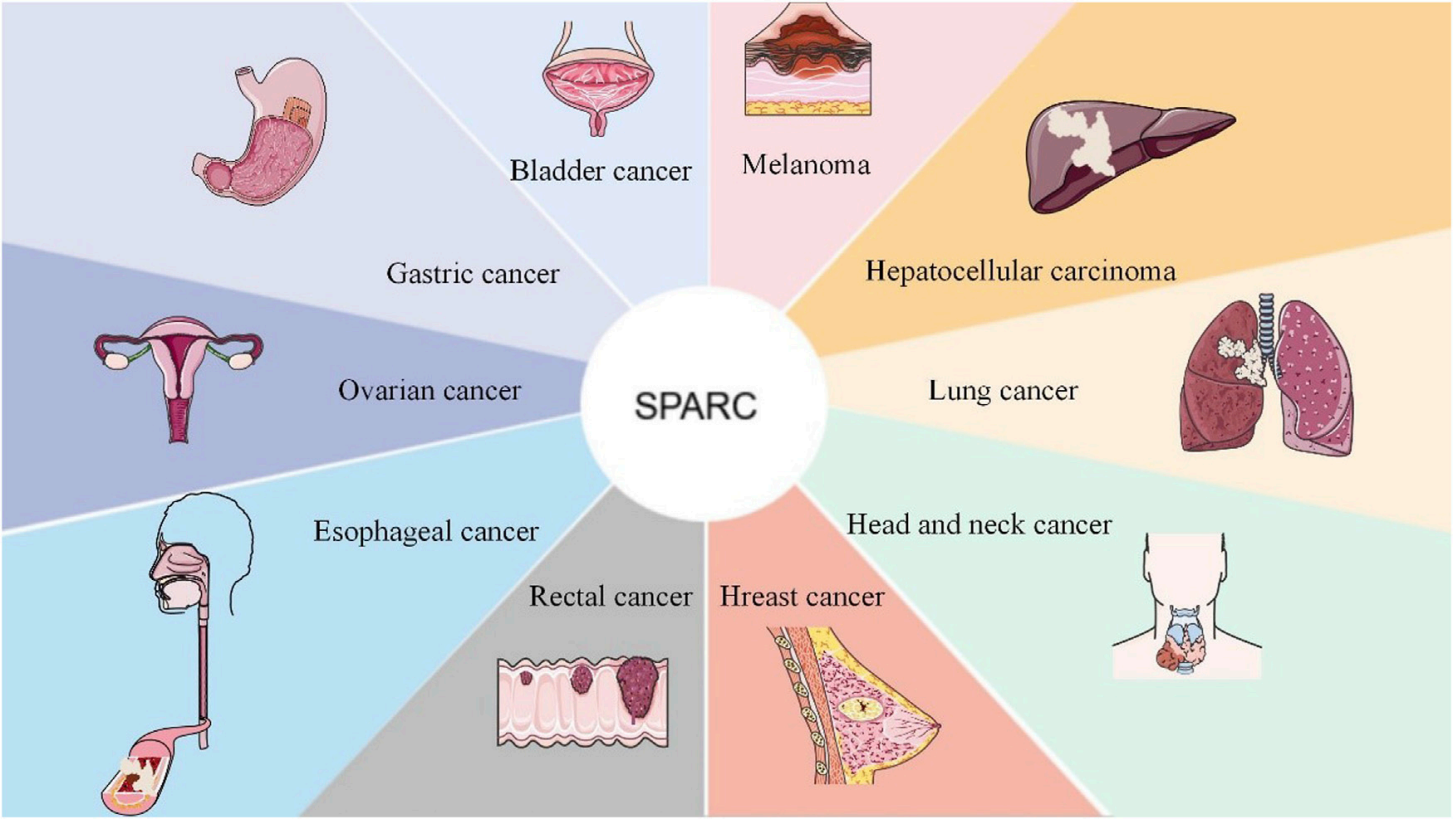
SPARC as a candidate target and as a therapeutic prognostic indicator for the adjuvant treatment of cancer. As a new therapeutic target, SPARC is extremely expressed in most tumors and will surely provide greater convenience for the effective treatment of tumors with functional nanomaterials in the future.

SPARC can be used as a prospective therapeutic index and prognostic biomarker for cancers including rectal cancer, bladder cancer, and head and neck cancer. SPARC was negatively correlated with tumor development. As a candidate therapeutic approach for tumors, future research can focus on the highly expressed sites of SPARC in cancer cells. As a prognostic indicator and potential target for various tumors, it is shown in Figure 5.

## Diabetes

SPARC is considered an important factor in diabetes (Kos and Wilding, 2010). SPARC-null mice are profoundly diabetic. The cross-sectional SPARC levels of women with gestational diabetes mellitus were greater than those of normal controls (Recinella et al., 2020). SPARC might serve as a promising and intriguing protein target for potential therapeutic interventions or as a biomarker to track the progression of diseases (Atorrasagasti et al., 2022). SPARC is found to be highly expressed in adipocytes, as well as in parenchymal and non-parenchymal hepatocytes, and pancreatic cells. SPARC suppresses adipogenesis and promotes insulin resistance. SPARC dramatically exacerbates diabetes in mice fed a high-fat diet (Atorrasagasti et al., 2019).

### Regulatory mechanism of SPARC in diabetes

SPARC is reduced in the islets from diabetic donors (Harries et al., 2013). The occurrence of diabetes-related kidney growth was related to the reduction of mRNA and protein of SPARC. In the pathogenesis of diabetes-associated renal growth, SPARC is involved in its pathogenesis (Gilbert et al., 1995). Diabetes-associated mesenteric vascular hypertrophy is relevant to increased SPARC expression in vessel walls (Jandeleit-Dahm et al., 2000). SPARC deficiency inhibits the rise in superoxide production elicited by diabetes stimulation, avoiding the induced hepatocyte damage (Aseer et al., 2017). SPARC downregulates RGS4 protein in pancreatic beta cells to enhance insulin secretion (Hu et al., 2020). MiR-29 directly targets SPARC and adversely modulates glucose metabolism by suppressing SPARC expression. SPARC could be an ideal target for treating diabetes (Song et al., 2018).

### Role of SPARC in diabetes

SPARC is significantly associated with inflammation in late pregnancy (Xu et al., 2013). SPARC is highly expressed in obesity and type 2 diabetes mellitus (T2DM), and levels of SPARC in plasma are relatively high in T2DM patients (Wu et al., 2011). For diabetic rats, SPARC is significantly upregulated in the liver and downregulated in the pancreas (Aseer et al., 2015). In the plasma of diabetic patients, the content of SPARC is extremely elevated (Lee et al., 2013). The serum SPARC level in the diabetic nephropathy group was the highest (Li L. et al., 2015). SPARC is highly expressed in the islets of NOD mice and is highest in the islets of young mice. SPARC identifies novel regulators of islet survival and *ß*-cell growth, a new target for treating diabetes (Ryall et al., 2014). Serum SPARC levels in T2DM patients who have coronary heart disease were elevated (Wang et al., 2015). SPARC expression correlates with fat mass in human adipose tissues (Kos et al., 2009). SPARC can be identified as potential central biomarkers for future drug development in the SD rat model of DMED (Wang Y. et al., 2022). In proliferative diabetic retinopathy (PDR), SPARC is highly expressed and can be a valid therapeutic target for disease treatment (Boneva et al., 2021).

As one of the diseases with the most complications, diabetes continues to plague lives. Functional nanomaterials manipulate SPARC to treat diabetes, which will definitely become a hot topic in the future.

## Glaucoma

Glaucoma, a disease affecting the optic neuron, is the second most prevalent cause of irreversible blindness. One of the primary hazardous factors for the development of glaucoma is elevated intraocular pressure (IOP) (Wallace et al., 2014). SPARC has been detected in different ocular tissues, including the cornea, ciliary epithelium, and other tissues.

### Regulatory mechanism of SPARC in glaucoma

The influence of SPARC on TGF-β2-mediated ocular hypertension offers a violent disease connection to primary open-angle glaucoma (POAG) pathogenesis. SPARC may be a curative target for certain eye diseases, for instance POAG (Scavelli et al., 2015). In the iris of PACG, SPARC was markedly elevated. By modifying the organization of the ECM to affect the biomechanical properties of the iris, SPARC could have a role in the generation of PACG (Chua et al., 2008). The lack of SPARC reduces intraocular pressure in a glaucoma mouse model (Wallace et al., 2015). The survival of postoperative surgical wounds was significantly improved in a SPARC-null mouse model of chronic glaucoma filtration (Seet et al., 2010). A key regulator of the TGF-β2-mediated ocular hypertension node is SPARC. By limiting the expression of collagen IV and fibronectin, loss of SPARC markedly attenuates the effect of TGF-β2 (Swaminathan et al., 2014). TGF-β2-mediated IOP elevation: SPARC may be a downstream regulator (Kang et al., 2013). Network pharmacology identified SPARC as a gene associated with glaucoma pathogenesis (Moazzeni et al., 2019). SPARC is involved in IOP dysregulation and is a potential therapeutic target (Rhee et al., 2009). The outflow of the aqueous humor across the TM pathway may be impaired by SPARC.

### Role of SPARC in glaucoma

SPARC has a major role in aqueous humor outflow across the trabecular meshwork (TM) (Fu et al., 2021). For the treatment of glaucoma, SPARC knockout has emerged as an attractive option (Seet et al., 2012). SPARC was significantly elevated in idiopathic open-angle glaucoma and angle-closure glaucoma. SPARC may be considered a potential target for treating glaucoma (NikhalaShree et al., 2019). SPARC levels in aqueous humor are prognostic factors for the trabeculectomy surgical outcome (Zhang Z. et al., 2018). SPARC remains a prospective index for the treatment of glaucoma (Kang et al., 2011). In comparison with the cataract group, the level of SPARC was evidently higher in the APAC group and was positively correlated with IOP (Wang J. et al., 2018). SPARC is extremely issued in the fetal cornea and TM (Carnes et al., 2018).

SPARC has become a good target for the treatment of a series of ophthalmic diseases, such as glaucoma, and will bring good news to patients in the future. Scarless treatment will be preferred by everyone in the future.

## Manipulation of SPARC by functional nanomaterials

**FIGURE 6:**
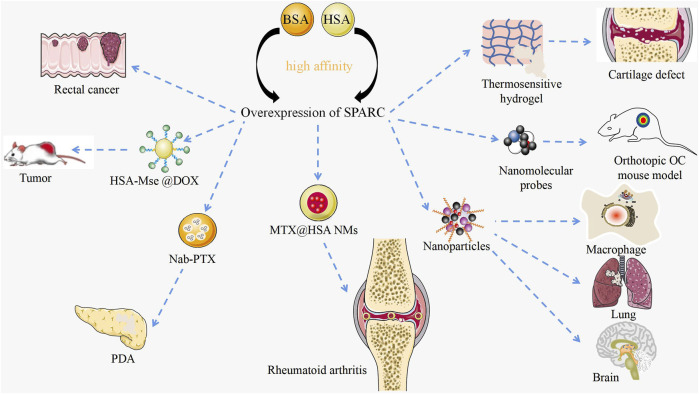
Targeted therapy with SPARC as a potential target. Current research on manipulating SPARC to treat diseases with novel nanomaterials. Functional nanomaterials can achieve targeted therapy of tumors by manipulating SPARC.

As a potential diagnostic marker and prognostic factor for disease, SPARC has achieved certain therapeutic results in its targeted agents as shown in Figure 6. The manipulation of SPARC by functional nanomaterials plays a role in further targeted treatment of diseases.

### Manipulation of SPARC by functional nanomaterials in RA and OA

RA and OA are bone degenerative diseases that have been plaguing clinics. Li et al. designed a thermosensitive hydrogel with negative charge targeting fibrocartilage for the continued supply of docetaxel to promote fibrocartilage clearing in a cartilage defect pattern. Fibrocartilage was stabilized by microtubule stabilization. The mechanism of clearing was confirmed to inhibit SPARC (Li J. et al., 2022). In RA and CIA joints, SPARC is highly overexpressed in the synovial membrane and synovial fluid. Given the major appetency between SPARC and HSA, MTX@HSA NMs have become effective potential nanomedicines for the cure of RA. Functional nanomaterial HSA might significantly improve the therapeutic effect and anti-inflammatory activity of MTX (Liu et al., 2019). Probes carrying functional nanomaterials determine the pathogenesis of OA by manipulating SPARC (Ceppi et al., 2019). RA and OA significantly affect the mobility of patients. Manipulating SPARC with functional nanomaterials to treat RA is bound to result in a series of problems.

### Manipulation of SPARC by functional nanomaterials in tumors

Dual-targeted therapy for metastatic rectal cancer was carried out by developing lactoferrin-mediated liposomes that bind to the LRP-1 receptor. Lactoferrin modification and endogenous albumin adsorption realize the advantages of functional nanomaterial manipulation of SPARC for rectal cancer (Xu Y. et al., 2022). A bioresponsive micro–nano (MTN) system was used for the effective clearance of *Cryptococcus neoformans in vivo.* The strategy is built on the excessive expression of matrix metalloproteinase 3 (MMP-3) in the infectious microenvironment (IME) and several associated target cells based on the overexpression of SPARC. Using BSA as a carrier, which is the natural carrier of SPARC, based on the overexpression of SPARC, NPs were deeper targeted to lung tissue, brain, and infected macrophages (Cheng et al., 2021). Albumin-based nanoparticles have been shown to be a useful drug delivery mechanism due to the intrinsic targeting of albumin through SPARC-mediated acceptor endocytosis (Hassanin and Elzoghby, 2020). Fibrotic stroma and tumor-promoting pancreatic stellate cells (PSCs) are key features in the microenvironment of pancreatic ductal adenocarcinoma (PDA). Nab-PTX is an HSA PTX nanoparticle. The enhanced matrix penetration may be due to its small *in vivo* size and the high affinity of HSA for SPARC (Wei et al., 2017). Based on the large avidity between HSA and SPARC, precise targeting of cancer sites can be achieved and the concentration of PDA drugs can be enhanced. HSA-coated MSe @ DOX forms redox-responsive nanoparticles (HSA-MSe @ DOX) on the surface, enhancing tumor targeting through nanoparticle interactions with SPARC in MCF-7 cells (Zhao et al., 2017). As a source of amino acids and energy for fast-growing cancer cells, SPARC is overexpressed in many tumors and its role is to transport albumin (Lin et al., 2016). Based on the high expression of SPARC in cancer and inflammatory sites, manipulating SPARC with functional nanomaterials to treat cancer will become an efficient and feasible strategy.

Using HSA or BSA as a carrier, the drug can precisely be targeted to the lesion, which avoids not only adverse reactions but also long treatment cycle and multiple biological disadvantages of side effects. The research of new nanomaterial manipulation on SPARC has gradually deepened.

## Future perspectives

The emergence of SPARC as a potential target and prognostic indicator is good news for the entire medical community. The investigation and research on the relationship between SPARC and cancer has been very comprehensive, but there are very limited articles on SPARC and RA for reference, which also brings limitations to the development and utilization of SPARC-targeted drug delivery systems. The effective treatment of RA has always been a difficult problem in the medical field, and the discovery of SPARC potential targets provides a bright spot for our future research. Summarizing the SPARC therapeutic mechanism and potential targets will provide impetus for the targeted drug delivery system controlled by functional nanomaterials or good prognosis of diseases.

## Summary

As an albumin-binding protein, SPARC was originally derived from human and fetal bovine bone and has high affinity with HSA and BSA. Multiple studies have found that SPARC is highly expressed in RA joint inflammation sites, various cancers, glaucoma, obese patients, diabetic patients, and melanoma patients as shown in Table 1. With the rapid development of the nano industry, targeted drug delivery systems are gradually emerging. Functional nanomaterials manipulate SPARC to produce anti-inflammatory therapeutic effects. RA and cancer are diseases that have plagued human beings for a long time. When alleviating the adverse reactions of patients, it is very critical to find a precise target for the effective treatment of cancer and RA. As a potential target for disease treatment, SPARC mostly uses HSA and BSA as carriers. When utilizing the affinity of the two, it can also efficiently and accurately load the drug for disease treatment into target cells. At present, based on several nanoparticles for the treatment of RA and tumors, for the treatment of RA, SPARC as a prospective target not only has an anti-inflammatory effect in the joint synovium but also realizes the precise targeting of drugs, achieving the effect of killing two birds with one stone.

**TABLE 1:** Summary of SPARC as a potential target and prognostic indicator.

Disease name	Mechanism	Reference
Rheumatoid arthritis	Negative regulation of NF-κB and TGF-β pathways	Mechanism of SPARC and SPARCL-1 regulating autophagy in glutamate-induced hippocampal neuron injury (xxx); Cheng et al. (2021); Critselis et al. (2019)
Gastric cancer	SPARC expression can promote gastric cancer cell invasion, metastasis, and angiogenesis	Haber et al. (2008); Hung et al. (2017)
Hepatocellular carcinoma	Overexpression of SPARC in HCC cells leads to reduced tumorigenicity in part by inducing MET	Ikuta et al. (2005)
Breast cancer	SPARC curbs the mobility and invasion of breast cancer, while resisting the platelet deficiency caused by SPARC	Lin et al. (2016); Lopez et al. (2006)
Non-small-cell lung cancer	By stimulating microvascular remodeling and excessive deposition of ECM proteins, SPARC induces pathological changes in non-small-cell lung cancer and idiopathic pulmonary fibrosis	Ng et al. (2013); NikhalaShree et al. (2019)
Melanoma	SPARC promotes cathepsin B-mediated melanoma invasion using collagen I and α2β1 integrin as mediators. SPARC helps in cell growth	Piñero et al. (2020); Said et al. (2007c)
Esophageal cancer	Downregulation of SPARC can reduce cell migration and invasion involved in EMT through the p-FAK/p-ERK pathway	Swaminathan et al. (2014); Termine et al. (1981)
Ovarian cancer	SPARC inhibits the NF-κB pathway mediating the macrophage-induced invasion of ovarian cancer cells	Wan et al. (2015); Wang et al. (2012)
Rectal cancer	Cox regression analysis identified the central genes of prognosis, and SPARC was the central gene	Wang et al. (2022a)
Bladder cancer	SPARC expression in human bladder cancer cell family negatively correlates with its proliferation rate and inhibits cell cycle progression by decelerating down the G1/S cell cycle	Wei et. (2017)
Head and neck cancer	SPARC strengthens cell proliferation, migration, and mesenchymal phenotype	Wu et al. (2017)
Diabetes	SPARC is expressed in adipocytes, parenchymal and non-parenchymal hepatocytes, and pancreatic cells. SPARC suppresses adipogenesis and promotes insulin resistance	Xu et al. (2022b); Xue et al. (2011); Ye (2017)
Glaucoma	The influence of SPARC on TGF-β2-mediated ocular hypertension gives a unique insight into the pathogenesis of glaucoma	Zhang et al. (2021); Zhao et al. (2022)

## Figures and Tables

**Table:** 

**SIN**	Sinomenine
**SPARC**	Secreted protein acidic and rich in cysteine
**NF-κB**	Nuclear factor-κB
**HSA**	Human serum albumin
**BSA**	Bovine serum albumin
**FLS**	Fibroblast-like synovial cells
**VEGF**	Vascular endothelial growth factor
**MMPs**	Matrix metalloproteinases
**FS**	Follistatin-like
**EC**	Extracellular
**ISG**	Interferon-stimulated gene
**RA**	Rheumatoid arthritis
**IHC**	Immunohistochemistry
**ELISA**	Enzyme-linked immunosorbent assay
**OA**	Osteoarthritis
**TGF-β**	Transforming growth factor-β
**ECM**	Extracellular matrix
**RANKL**	Receptor activator of nuclear factor-κB ligand
**GC**	Gastric cancer
**DEGs**	Differentially expressed genes
**PPI**	Protein–protein interaction
**GDM**	Gestational diabetes mellitus
**ADR**	Doxorubicin
**5-FU**	5-Fluorouracil
**DEGs**	Differentially expressed genes
**TME**	Tumor microenvironment
**PPI**	Protein–protein interaction
**GEO**	Gene Expression Omnibus
**MET**	Mesenchymal–epithelial transition
**ROS**	Reactive oxygen species
**LDH**	Lactate dehydrogenase
**NAFLD**	Non-alcoholic fatty liver disease
**AMO**	Anti-miR-29a oligonucleotide
**NCRT**	Neoadjuvant chemoradiotherapy
**SNPs**	Single-nucleotide polymorphisms
**HSCs**	Hepatic stellate cells
**TNBC**	Triple-negative breast cancer
**IRS**	Immune response scoring
**MDSCs**	Myeloid-derived suppressor cells
**CTGF**	Connective tissue growth factor
**PT**	Phyllodes tumors
**ISH**	*In situ* hybridization
**NSCLC**	Non-small-cell lung cancer
**ET-3**	Endothelin-3
**EMM**	Epidermal melanoma metastases
**CSM**	Common skin metastasis
**ESCC**	Esophageal squamous cell carcinoma
**FFPE**	Formalin-fixed paraffin-embedded
**BE**	Barrett^’^s esophagus
**siRNA**	Small interfering RNA
**OvCa**	Ovarian cancer
**LARC**	Locally advanced rectal cancer
**T2DM**	Type 2 diabetes mellitus
**NOD**	Non-obese diabetic
**SAAT**	Subcutaneous abdominal adipose tissue
**DME**D	Diabetic erectile dysfunction
**TM**	Trabecular meshwork
